# Supplementary material for: TCEPVDB: Artificial Intelligence-Based Proteome-Wide Screening of Antigens and Linear T-Cell Epitopes in the Poxviruses and the Development of a Repository
Source: Proteomes. 2025 Nov 6;13(4):58. doi: 10.3390/proteomes13040058 (PMC12642008; doi:10.3390/proteomes13040058)
Supplement: Supplementary file 1 [file proteomes-13-00058-s001.zip › proteomes-3834123-supplementary.pdf]

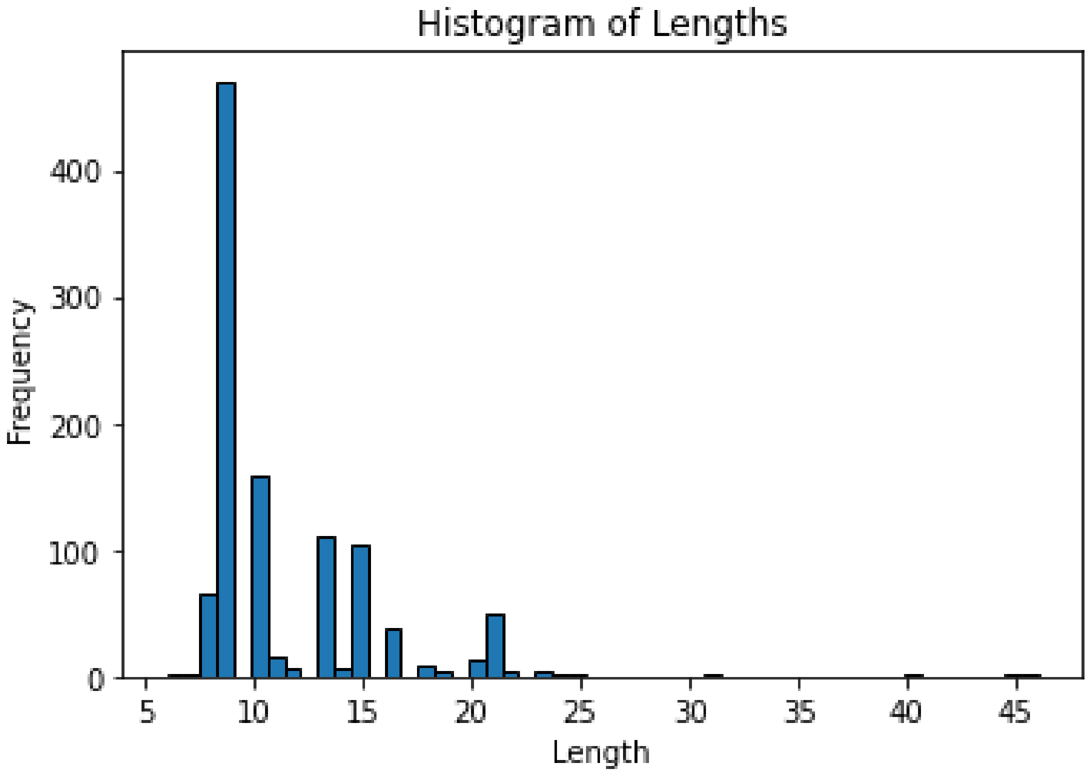

Supplementary Figure S1 – Length distribution of the T Cell epitopes used for training the PoxiPred model. We considered all the 1,067 T-cell epitopes that were used in the training process of PoxiPred [17]. The distribution of the lengths is plotted. The 25th and 57th percent quartile of the length of the epitopes range from 9 to 13 amino acids of length, respectively.
